# The Considerations for the Prescription of Valproate as Treatment in Mayo Mental Health Service

**DOI:** 10.1192/bjo.2025.10189

**Published:** 2025-06-20

**Authors:** Irene Akashie, Rachael Ayodele, Philippa Renton, Sharon Coen, Catherine Kelly

**Affiliations:** Mayo Mental Health Services, Castlebar, Ireland

## Abstract

**Aims:** To examine current monitoring practices for valproate users within Mayo Mental Health Services and analyse the conditions for which it is prescribed.

**Methods:** Information on sodium valproate users was collected from clinical records using a data collection tool specifically developed to capture indicative parameters.

**Results:** Data was collected from 6 community clinics and 4 inpatient units with a total of 69 patients on sodium valproate treatment within the service. Licensed indication was accounted for in 24.6% of patients with 63.8% prescribed valproate for unlicensed use and 11.6% for unknown indication. Amongst the prescribed users 33.3% were male and 66.7% were female with 27.5% of participants being under 55 years old and 72.5% over 55 years old. Out of 39 females using valproate 20.5% were on effective contraceptives and 79.5% were not using any. Regarding the annual physical health check, all participants in the approved inpatient units were compliant as it was part of the inpatient care requirement. 54.5% of patients in the community clinic on valproate had annual physical health reviews and 45.5% were not compliant.

**Conclusion:** Our examination of Mayo Mental Health Service shows that most valproate use was for unlicensed indications with the majority of the users being female, most of whom were over 55 years of age. This reflects the high percentage of non-effective contraceptive users. There is also inadequate physical health monitoring for patients in the community.

